# Perturbation of invadolysin disrupts cell migration in zebrafish (*Danio rerio*)

**DOI:** 10.1016/j.yexcr.2013.02.005

**Published:** 2013-05-01

**Authors:** Sharron Vass, Margarete M.S. Heck

**Affiliations:** University of Edinburgh, Queen's Medical Research Institute, University/BHF Centre for Cardiovascular Science, 47 Little France Crescent, Edinburgh EH16 4TJ, UK

**Keywords:** Invadolysin, Metalloprotease, Cell migration, Vascular development, Zebrafish

## Abstract

Invadolysin is an essential, conserved metalloprotease which links cell division with cell migration and is intriguingly associated with lipid droplets. In this work we examine the expression pattern, protein localisation and gross anatomical consequences of depleting invadolysin in the teleost *Danio rerio*. We observe that invadolysin plays a significant role in cell migration during development. When invadolysin is depleted by targeted morpholino injection, the appropriate deposition of neuromast clusters and distribution of melanophores are both disrupted. We also observe that blood vessels generated via angiogenesis are affected in invadolysin morphant fish while those formed by vasculogenesis appear normal, demonstrating an unanticipated role for invadolysin in vessel formation. Our results thus highlight a common feature shared by, and a requirement for invadolysin in, these distinct morphological events dependent on cell migration.

## Highlights

► Invadolysin is a conserved metalloprotease linking cell division and migration. ► Morpholino-induced knockdown of invadolysin perturbs cell migration in zebrafish. ► Specifically, neuromast deposition and melanophore migration are disrupted. ► Additionally, angiogenic formation of blood vessels is perturbed in early embryos. ► All observed phenotypes can be rescued by co-expression of wild type transcript.

## Introduction

The proteins that control cell migration are vital not only for normal health and development, but are also frequently implicated in the progression of disease. It is fundamentally necessary for cells to be able to move from one place to another when an organism is developing, e.g. this process is required for nervous and vascular system development, and for appropriate responses to immune challenges. However, when the mechanisms that control cell migration go awry, the consequences can be catastrophic, as when cancer cells become metastatic or endothelial cells form vasculature inappropriately.

Invadolysin was originally identified in a screen designed to discover mutations affecting mitosis and higher order chromosome structure in *Drosophila melanogaster*. Its drastic effects on cell cycle dynamics, chromosome structure, nuclear envelope protein accumulation in mitotically active tissues, and germ cell migration in developing embryos are thought to be the cause of late larval lethality [1]. The gene belongs to the leishmanolysin class of proteases [2] and encodes a conserved zinc metalloprotease that represents the metazoan counterpart of the M8 family of metzincins. The name *invadolysin* also reflects the localisation to structures resembling invadopodia.

Invadolysin is conserved from bacteria to plants and higher vertebrates and is phylogenetically distinct from the Matrix MetalloProtease (MMP) and A Disintegrin And Metalloprotease (ADAM) families [3]. We have identified one variant of invadolysin in fruit flies, four alternatively-spliced forms in human cells, and the zebrafish contain two splice variants of this gene. Analysis in a number of human cell lines has shown that invadolysin is associated with lipid droplets [4], and that *Drosophila* lacking the invadolysin protein also contain less triglycerides.

In order to gain a better understanding of invadolysin function in a vertebrate, we decided to analyse this novel metalloprotease in *Danio rerio* (zebrafish). Zebrafish presents an excellent system in which to study gene function in a developing vertebrate, and has been used extensively in genetic screens and developmental studies [5,6]. Furthermore, this model system has been used to analyse many diverse regenerative processes including retinal development [7], those following spinal chord crush injuries [8], and more recently, heart regeneration [9].

The development of gene-specific morpholino oligonucleotides (MO) has facilitated the analysis of depletion, or knock-down, phenotypes in both zebrafish and *Xenopus* embryonic development [10–12]. In this study, we have capitalised on the use of MO-induced blockade of translation and GFP/dsRed reporter lines to analyse the in vivo consequences of depleting invadolysin. We observed a reduction in the number of mechanosensory cell clusters known as neuromasts, defects in melanophore migration, and inhibition of blood vessel formation through angiogenesis. As already demonstrated in *Drosophila*, these results lend additional support to a role for invadolysin in cell migration, and further extend this function to the likely involvement of particular vertebrate chemokine signalling pathways.

## Materials and methods

### Ethics statement

All experiments were approved by the local ethics committee and conducted in accordance with the Animals (Scientific Procedures) Act 1986 in a UK Home Office approved establishment. Under these guidelines no specific ethical approval is required for work on zebrafish younger than 120 hpf (hours post fertilisation).

### ***Danio rerio*****Husbandry**

Zebrafish (*Danio rerio*) of the WIK and AB strains were raised and maintained under constant temperature of 28 °C, a 14 h light/10 h dark cycle, and fed on *Artemia*. Embryos were collected using marble filled containers and staged according to [13] or by hpf. *Tg*(*flk*1:*GFPnls*;*GATA*1:*dsRed*) line was a kind gift from Dr. Tim Chico, University of Sheffield.

### Morpholino sequences

The following anti-sense morpholino oligonucleotides (obtained from Gene Tools LLC, Philomath, USA) were generated to target the initiating ATG of zebrafish LMLN (XM_684004). ATG-MO: 5′ GAG GCT GCC GCC ATC CTG ACG CCA T 3′ [fluorescein]. Con-MO: 5′ GAc GCT cCC GCg ATC CTG AgG CgA T 3′ [fluorescein]. The Con-MO oligo contains 5 mis-matched base pairs (lower case) and served as a toxicity and phenotype control. The Ex6-MO morpholino is designed to target the exon/intron boundary at the 3′ end of Exon 6: 5′-TCC TTA AAT ATC TCC GTT ACC TGT C-3′ [lissamine rhodamine]. The oligos were manufactured to incorporate either a 3′ fluorescein or lissamine rhodamine moiety to aid visualisation of morpholino uptake in the developing embryo. All morpholinos were re-suspended in dH_2_O to a concentration of 1 mM and stored at −80 °C.

### Morpholino micro-injection

Prior to injection, the morpholino oligos were diluted to the appropriate concentration in dH_2_O. 0.1% aqueous phenol red was added to assist visualisation of injected bolus. A bolus of approximately 5 nL was injected into freshly collected embryos at the 1–2 cell stage. A range of concentrations was used to establish the best survival to phenotype ratio. In this study, a concentration of 100 nM was found to be optimal, thus the injected dose was approximately 5 ng. Embryos were aligned in rows on a plastic plate and injected using a micro-manipulator and micro-injector. The injected embryos were washed into a petri-dish with salted system water (conductivity 400 microsiemens) containing 0.1% methylene blue, and allowed to develop in a 28 °C incubator.

### 4-Di-2-asp staining

4-(4-(Diethylamino)styryl)-*N*-methylpyridinium iodide (4-Di-2-Asp, Sigma) was used to visualise neuromast clusters. A 1:1000 dilution of 5 mg/ml stock in dH_2_O was added to 35 mm petri-dishes containing fish of the appropriate stage. The fish were allowed to swim freely in the solution for 10 min. Anaesthetic (MS-222) was added after staining, the neuromast cells were then visualised using 460 nm light on a Leica ZX microscope and photographed using a Leica RFX camera.

### Immunofluorescence and live imaging

All immunofluorescence staining was carried out as follows: anaesthetised fish of the appropriate stage were placed in fresh fixation buffer (PBS+4% formaldehyde+2% Triton X-100) overnight at 4 °C. The fish were washed 3×5 min with PBS+0.1% Triton X-100 (PBS-Tx) and then blocked in PBS-Tx+3% BSA for 1–2 h at room temperature. Fish were washed 2×5 min in PBS-Tx, and the primary antibodies (diluted in PBS-Tx+0.3% BSA) were added, then left to incubate overnight at 4 °C. The fish were then washed 3×5 min in PBS-Tx and the secondary antibodies (plus 0.5 μg/ml of DAPI if using) were added (diluted in PBS-Tx) and incubated at room temperature for 2 h. The fish were then washed 4×5 min in PBS-Tx, and finally mounted in PBS containing 50% glycerol.

The primary antibodies used in this work include: two custom made rabbit anti-zebrafish LMLN antibodies (A6970 and A6971) raised against peptide sequence YCDSVRSAPLQLTC (Genosphere Biotechnologies) which were used at 1:250 dilution, and mouse anti-acetylated tubulin clone 6–11 B-1 (T6796, Sigma) used at 1:500 dilution. Secondary antibodies were diluted at 1:500 and include: Alexa Fluor 594 donkey anti-rabbit IgG (A21206), Alexa Fluor 488 donkey anti-mouse IgG (A21202), and Alexa Fluor 488 Phalloidin (A12379)—all from Invitrogen. DAPI was used at 0.5 μg/ml. Control experiments were performed using pre-immune sera and secondary antibody alone. The staining patterns shown herein were not observed in control experiments.

Slides were viewed on an Olympus Provis microscope, equipped with epifluorescence optics. Images were captured using an Orca II CCD camera (Hamamatsu) and SmartCapture 2 software (Digital Scientific). The resulting files were processed using Adobe Photoshop.

Fish for live imaging were anaesthetised with 4.2% MS-222, then immobilised in 3% methylcellulose containing anaesthetic. Imaging was carried out using a Leica SP5 scanning confocal microscope. 3D max projections were obtained using the LAS-AF software. AVI files and single images were produced in Image J.

### Sub-cloning of zebrafish invadolysin

Total RNA was extracted from 96 hpf zebrafish embryos using the QIAGEN RNeasy mini kit following the manufacturer's instructions. Additional homogenisation was required during the initial tissue lysis step; this was done on ice using a small-motorised pestle. cDNA was generated using random hexamers and SuperScript III reverse transcriptase (Invitrogen) as per the manufacturer's instructions. PCR amplification of full-length invadolysin was carried out using *Pfu* polymerase (Promega) and primers to the 5′ and 3′ UTRs which incorporated an EcoR1 site (5′ UTR-CGAATTC GAC GGA GAT ATG AAA GAT AAA GCA C and 3′ UTR-CGAATTC CCC TCC ACG TCA GCT AAA CC), under the following conditions: 95 °C 2 min, 95 °C 1 min, 62 °C 3 min, 74 °C 4 min, hold at 74 °C for 10 min. The resulting product was purified using QIAquick gel extraction kit, digested with EcoR1, ligated into pre-digested CIP-treated pBS KS (+) using T4 DNA ligase (Promega), and electroporated into JM109 cells. Candidate colonies were selected, grown overnight in LB with antibiotic and DNA extracted using the QIAGEN mini-prep kit. Prospective clones were screened by restriction digest. 5′ 7-methylguanylate capped RNA was synthesised using mMESSAGE mMACHINE kit (Ambion) according to the manufacturer's instructions.

### Tissue lysis and immunoblotting

Anaesthetised fish were placed in PBS and 3X Sample Buffer (2% SDS, 50 mM Tris pH 6.8, 10% glycerol, 0.1% Bromo-phenol Blue, 2 mM EDTA) and 0.1 M DTT. The fish were homogenised by hand for 10 s, then the samples were briefly sonicated at 5–10% amplitude and boiled for 2 min. Samples were stored at −20 °C until required. Protein samples were resolved by SDS-PAGE on 4–12% bis-tris pre-cast gels (Novex), transferred to nitrocellulose filter membranes, and processed for immunoblotting as described above.

Proteins resolved by SDS-PAGE were transferred to Protran™ nitrocellulose filter membrane (Schleicher & Schuell) in Towbin Buffer (192 mM Glycine, 25 mM Tris (Sigma 7–9), 20% Methanol, 0.1% SDS) for 3 h, 300 mA at 4 °C. The transferred proteins were visualised by staining the filter membrane with Ponceau S. The filter membranes were washed in 1X PBS plus 0.1% Tween20 (PBS-Tw), blocked with 5% w/v dried milk powder (Sainsbury's) in PBS-Tw, washed in PBS-Tw and incubated with appropriate primary antibody in PBS-Tw for 2 h at RT or overnight at 4 °C. The filter membranes were then washed 2×5 min, 1×15 min and 2×5 min in PBS-Tw and then an appropriate horse radish peroxidase (HRP) conjugated secondary antibody in PBS-Tw was applied for 60 min at RT. The filter membrane was then washed 2×5 min, 1×15 min and 2×5 min in PBS-Tw. Excess wash solution was drained off and equal volumes of enhanced chemiluminescence (ECL) solutions (GE Healthcare) were added for 1 min, then the filter membrane was exposed to Kodak XAR-5 film (Sigma) before developing.

Control experiments were performed using pre-immune sera and secondary antibody alone. The banding patterns shown herein were not observed in the control immunoblots at similar dilutions or exposures.

## Results

### Zebrafish invadolysin contains a conserved metalloprotease motif

**Fig. 1:**
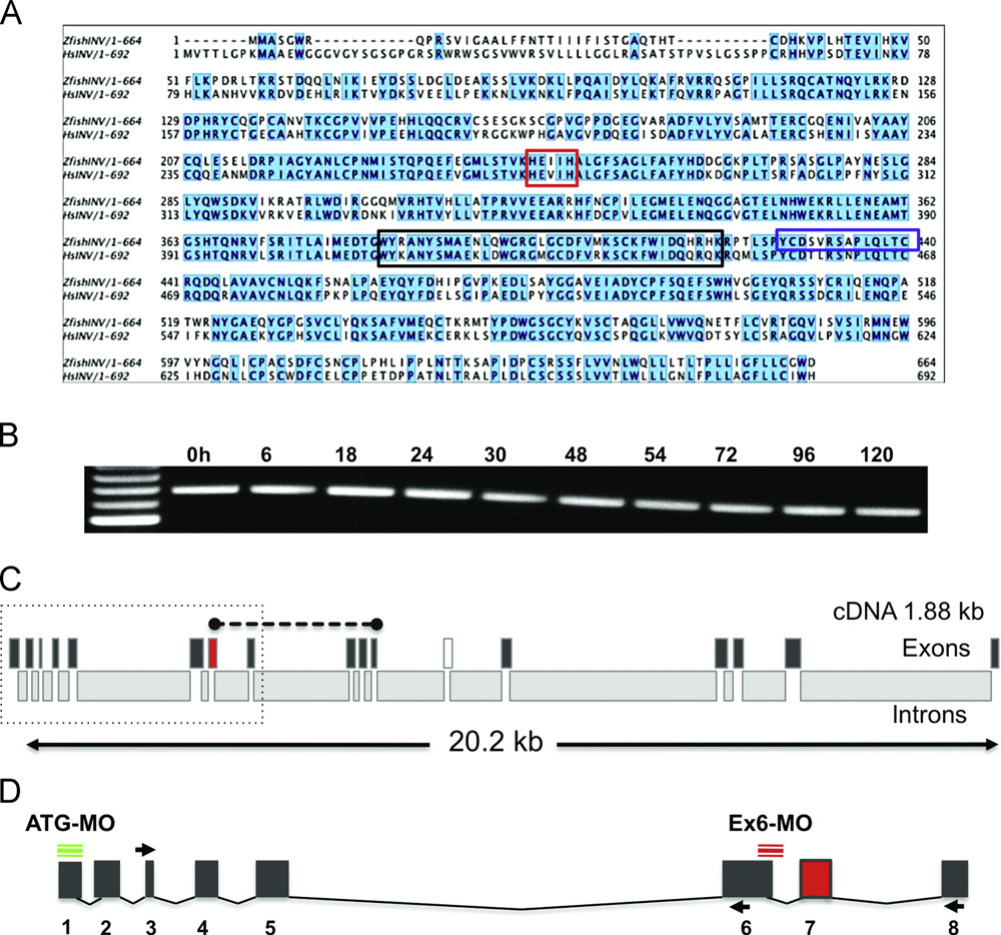
Invadolysin is a conserved metalloprotease. (A) Jalview alignment of zebrafish and human invadolysin open reading frames showing 69% identity (blue shading). The conserved proteolytic domain is outlined in red, the alternatively spliced exon in black, and the peptide for antibody production in blue. (B) RT-PCR analysis of cDNA obtained from different time points during the first 120 h of development, primers designed to span exon 12. (C) Schematic representation of exon/intron boundaries in zebrafish Invadolysin. The exon containing the catalytic motif is shown in red, the alternatively spliced exon in white. The dashed line denotes an invadolysin-specific ribo-probe. The dotted box indicates the region of interest magnified in panel D. (D) Enlargement of Exon 1–8 showing targeting position of morpholino oligos (green and red) and primers (arrows) used for RT-PCR.

Invadolysin is a conserved zinc-metallopeptidase of the M8 superfamily. We have identified one splice variant in *Drosophila*, four in humans, and two in zebrafish. The predominant splice variant in human cells, as detected by RT-PCR analysis, is the v1+37 form (with an unprocessed size of 692 aa) [4], which shares 69% identity with the *Danio* open reading frame (664 aa) when aligned using Jalview [14] (Fig. 1A). The unprocessed *Drosophila* open reading frame is 683 aa long and shares 43% identity with the *Danio* sequence. (The human and fly forms share 44% identity.) In zebrafish, the two variants differ (as in humans) by the alternative splicing of an exon encoding 37 amino acids (Fig. 1A, black box). The splice variant including this exon is however the more prevalent form expressed during the first 120 h of development (Fig. 1B).

Predicted proteolytic activity is attributed to the conserved metzincin active site residues ‘HEXXH’, located in exon 7 (Fig. 1A, red box). This sequence has been shown to form part of the zinc-binding site in crystallographic studies of leishmanolysin, and is essential for the catalytic activity of this family of metallopeptidases [15,16]. In zebrafish, there is only one copy of the gene, located on chromosome 9 (gene identifier: 560606, protein accession number: XP_689096). The gene spans 20.2 kb and is encoded by 17 exons (Fig. 1C). We generated a gene specific ribo-probe (Fig. 1C, dashed line) and peptide antibodies (Fig. 1A, blue box) to examine the expression pattern and protein localisation during the first 120 h of development.

### Invadolysin is localised to distinct structures in developing embryos

**Fig. 2:**
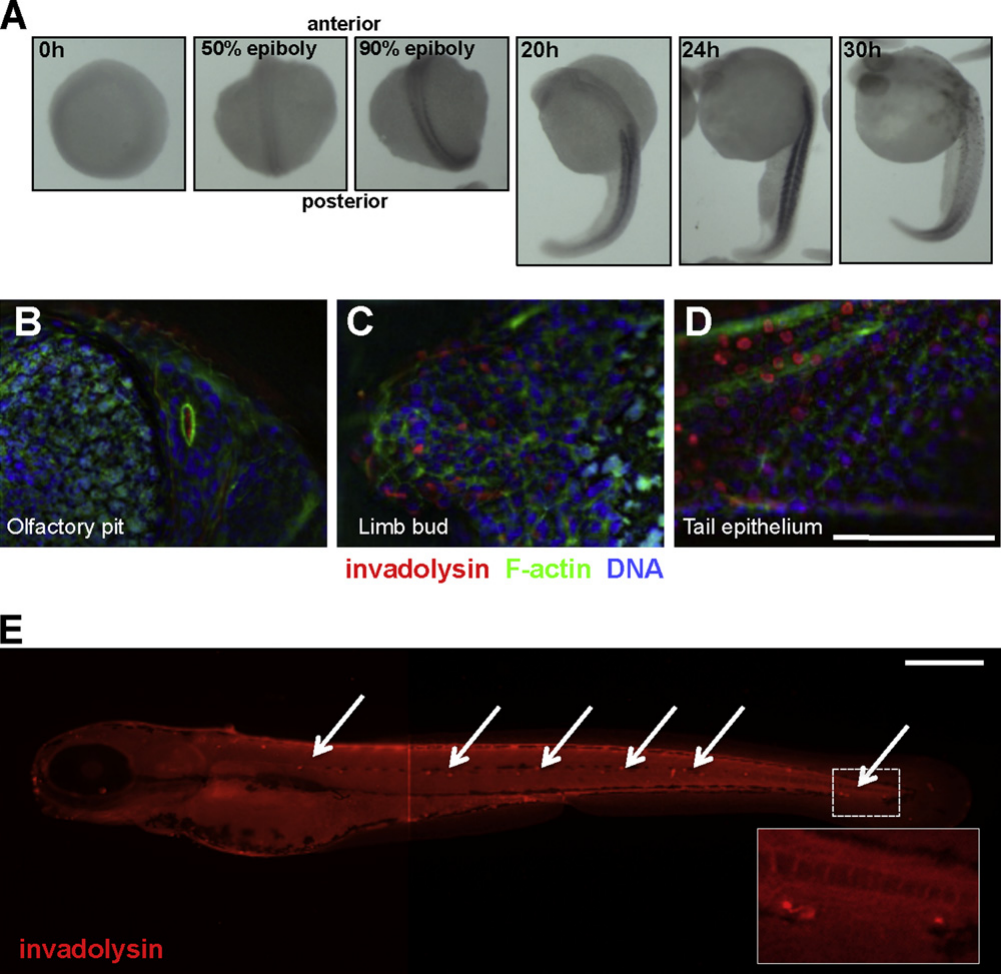
Staining of embryos with antibodies to zebrafish invadolysin. (A) RNA in situ hybridisation using an invadolysin specific anti-sense ribo-probe in 0–30 hpf zebrafish embryos. Scale bar=250 μm. Immunofluorescence images of the (B) cilia of the developing olfactory system, (C) a limb bud and, (D) tail epithelium section of a 48 hpf embryo. Invadolysin (red), F-actin (green), and DNA (blue). (E) Invadolysin antibody staining of neuromast cells (white arrows) in a 72 hpf embryo. Inset panel: enlargement of region in dashed box. Scale bar=250 μm.

Expression of the invadolysin transcript is not detectable in 0–30 min embryos, but is observed, albeit faintly, by RNA *in situ* hybridisation in early gastrula stage embryos around 50% epiboly (Fig. 2A). By 90% epiboly, invadolysin expression is detected as two distinct stripes, potentially in the developing somites. We observed this somite-like expression pattern to move caudally in the developing tail until approximately 30 hpf. However, we could detect no localised RNA expression pattern at the gross anatomical level after this developmental stage.

To examine the localisation of invadolysin protein, fish embryos were collected, fixed and processed for immunofluorescence at various time points. We generated two rabbit polyclonal antibodies to a zebrafish invadolysin peptide chosen with the ‘antigenic epitope’ prediction software from EMBOSS (Fig. 1A, blue box) [18]. Both antibodies revealed identical staining patterns, which were not observed with pre-immune serum. Fig. 2(B–D show the localisation of invadolysin (red) in the olfactory pit, the developing limb bud, and tail fin of a 48 hpf larva. At the later time point of 72 hpf, we observed the protein to be associated with specific punctate structures along the lateral line of the developing fish (Fig. 2E). By utilising 4-Di-2-Asp: 4-(4-(Diethylamino)styryl)-*N*-methylpyridinium iodide, a cationic specific dye that binds to mitochondria of live cells (including hair cells), we were able to demonstrate that these structures represented neuromast clusters.

Neuromasts are a collection of cells that comprise the mechanosensory organs required for environmental sensing, which is necessary for schooling behaviour and predator avoidance (for a review, see [19]). Neuromasts arise from a small group of primordial cells that migrate from the otic placode either rostrally to form the anterior lateral line system of the head, or caudally to form the posterior lateral line. This process is initiated at 18–20 hpf, and is normally complete by 48 hpf. As the primordium migrates, it deposits small clusters of approximately 20 pro-neuromast cells that then differentiate into three cell types including mechanosensory hair cells, support cells and mantle cells [17,20].

In order to gain an understanding of invadolysin's function during early vertebrate development, we chose to analyse the effect of protein knock-down using targeted morpholino antisense oligonucleotides. We generated 3 morpholinos to target the invadolysin transcript. The first was designed to target the initiating methionine codon and is referred to as ATG-MO (Fig. 1D). The second morpholino, referred to as Con-MO (control), was also designed to target the initiating methionine but contained 5 mismatched bases relative to the original target sequence. This mismatched morpholino serves as an indicator of MO toxicity and mechanical injury. Both the ATG-MO and the Con-MO contain a fluorescein moiety to enable detection by epifluorescence microscopy. The third morpholino (Ex6-MO) was designed to interrupt the splicing of the exon 6-3′/5′-intron boundary (located upstream of the proteolytic domain) and contained a lissamine rhodamine tag (Fig. 1D).

**Fig. 3:**
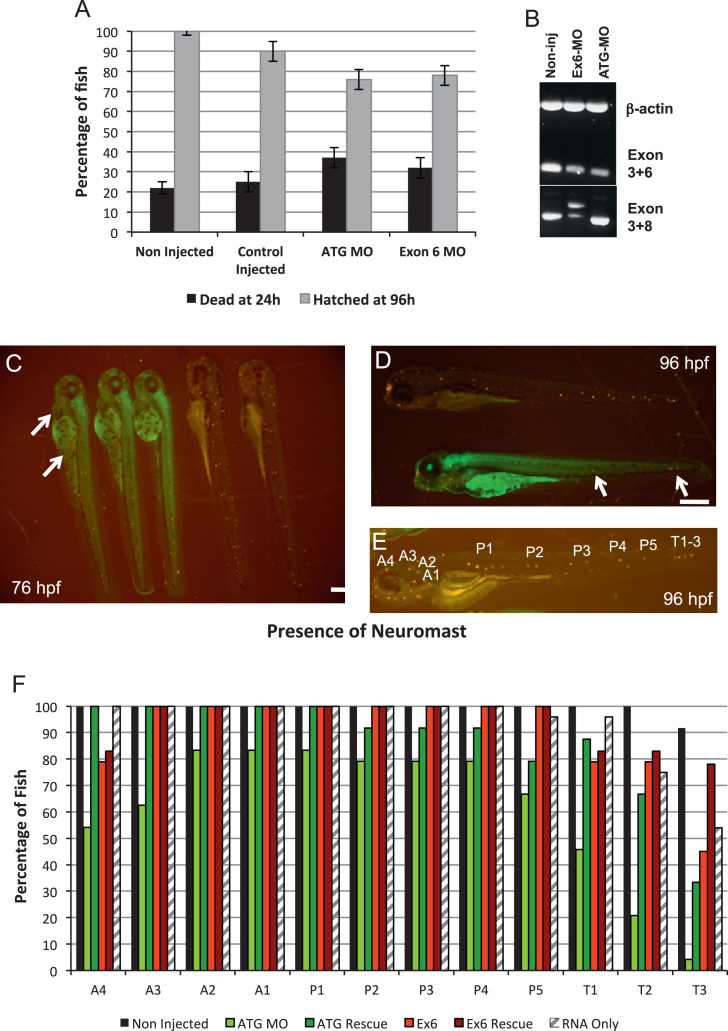
Morpholino induced knock-down of invadolysin affects fish viability and results in a decreased number of neuromast clusters. (A) Graph comparing mortality at 24 hpf (black bars) and hatching by 96 hpf (grey bars) in non-injected, Con-MO, ATG-MO and Ex6-MO injected fish. The data were obtained from 3 experiments, with a minimum of 100 fish per sample. (B) RT-PCR analysis of cDNA obtained from non-injected, Ex6-MO, and ATG-MO injected 72 hpf embryos. Primers were designed to detect *β*-actin (250 bp), invadolysin exons 3–6 (250 bp), and invadolysin exons 3–8 (510 bp). An aberrant, larger splice product was observed with the primers flanking exons 3 and 8 in the larvae injected with the Ex6-MO. (C) 4-Di-2-Asp staining in 76 hpf ATG-MO (left) and non-injected (right) embryos. Arrows indicate aberrant melanophore distribution and pericardial oedema. (D) 4-Di-2-Asp staining in 96 hpf non-injected (top) and ATG-MO (bottom) injected embryos. Fewer neuromast clusters were observed in the ATG-MO injected fish (arrows). Scale bar=250 μm. (E) 4-Di-2-Asp staining in 96 hpf non-injected fish (longer exposure) showing specific clusters that were scored. (F) Comparison of neuromast clusters scored in non-injected, ATG-MO, Ex6-MO, invadolysin transcript injected, and rescued 76 hpf embryos. Non-injected samples (black), ATG-MO (green), ATG-MO+transcript (dark green) Ex6-MO (red) Ex6-MO+transcript (dark red) invadolysin transcript only (striped). A minimum of 24 fish were scored for each sample.

Freshly collected wild type embryos were injected at the 2–4 cell stage with a range of morpholino dilutions to establish which concentration gave the highest phenotype to survival ratio. We observed that a morpholino concentration of 100 nM (an injected amount of approximately 5 pg) resulted in an average 32–37% mortality rate for both ATG-MO and Ex6-MO at 24 hpf, compared to 25% for the Con-MO and 20% for the non-injected fish (Fig. 3A). Higher morpholino concentrations resulted in an increased mortality and the appearance of highly deformed embryos in the Con-MO injected group.

Fish that were injected with MOs were observed on a daily basis up to 120 hpf and all resulting phenotypes were scored. The fish injected with the ATG-MO fell into 3 distinct categories, which appeared directly proportional to the amount of incorporated morpholino as visualised by fluorescence microscopy. The brightest fish often displayed severe developmental abnormalities including kinked tails, yolk sac oedema**,** failure of eye development and shorter body length. These fish often did not survive past 24 hpf, whereas the least fluorescent fish appeared to develop relatively normally. The majority of fish fell into a middle category exhibiting a moderate level of fluorescence, and displaying a range of phenotypes including pericardial oedema, lack of motility, and an impaired hatching at 96 hpf. Fish injected with the Con-MO exhibited similar survival and hatching rates to the non-injected cohort, while fish that were injected with the Ex6-MO displayed similar phenotypes and mortality to those injected with the ATG-MO (Fig. 3C), suggesting these phenotypes were due to effects on invadolysin.

The molecular consequence of the Ex6 splice-interrupting MO was analysed by RT-PCR. This MO contains 5 nucleotides of exon sequence and 20 bases of intronic sequence and as such would be anticipated to prevent the normal spice event from occurring. This may in turn, result in an abnormal transcript, incorporating part of the intron into the resulting mRNA [21]. Primers detecting *β*-actin were used as a control, whereas a primer combination to detect invadolysin cDNA (Fig. 1D, between exons 3 and 6) was used to analyse invadolysin in the different samples. A primer combination detecting the cDNA between exon 3 and exon 8 (Fig. 1D) was used to verify whether Ex6-MO affected splicing. When mRNA from 72 hpf non-injected, ATG-MO and Ex6-MO injected fish was analysed by RT-PCR, each sample generated the expected PCR product with the various primer combinations used (Figs. 1D and 3B). In addition, while the predicted product was present upon Ex6-MO injection, a larger product was also apparent, which we interpret to represent a mis-spliced product (Fig. 3B).

### Invadolysin is required for appropriate formation of neuromast clusters

In order to quantify the number of neuromast clusters in non-injected, Con-MO and ATG-MO injected samples, fish were examined at daily time points after staining with 4-Di-2-Asp. Lateral line progression towards the tail is normally complete by 30 hpf, however, the terminal neuromasts take between 48–96 hpf to form and become visible by 4-Di-2-Asp staining. For the purposes of quantification, we counted neuromasts at 76 and 96 hpf (Fig. 3C and D). Direct comparison between a non-injected (top) and morphant (bottom) fish at 96 h show that although the terminal neuromasts are in place on the morphant fish, a number of these structures are absent along the trunk (Fig. 3D). Fish injected with the Con-MO did not differ significantly from the non-injected samples.

The number of neuromasts at specific anterior or posterior positions (Fig. 3E) was scored in 76 hpf non-injected, ATG-MO and Ex6-MO injected fish (Fig. 3F). In 76 hpf non-injected fish, neuromasts were present at 100% of the body locations scored, with the exception of the three terminal neuromast clusters, which were observed in 96% of cases (black bars). In contrast, 76 hpf ATG-MO injected fish showed a marked reduction in the number of neuromasts at all body locations scored, most significantly at the anterior and posterior terminal positions (green bars). Those fish injected with the Ex6-MO also displayed a marked reduction in the number of terminal neuromasts (red bars), but the effect was not as dramatic as that observed with ATG-MO.

In addition to scoring the number of neuromasts in non-injected, ATG-MO and Ex6-MO morphants, we also injected 5′ 7-methylguanylate capped full-length invadolysin transcript either alone or in conjunction with both the ATG-MO and Ex6-MO, with the aim of rescuing the morphant phenotype. With the exception of the terminal neuromasts, the injection of invadolysin transcript alone did not alter the number of neuromasts at the other positions scored in comparison to non-injected fish (striped bars). However, when fish were co-injected with ATG-MO and full-length invadolysin transcript, significantly more neuromasts were present than in the ATG-MO alone injected fish (compare green with dark green bars), suggesting that the injection of invadolysin transcript at least partially rescued the deleterious effects of the ATG-MO on neuromast formation. A similar effect was observed upon co-injection of Ex6-MO and full-length invadolysin transcript (compare red with dark red bars).

Statistical significance for comparison of neuromast scores between groups at 76 hpf was analysed by performing a Kruskal–Wallis one-way analysis of variance test with Dunn’s multiple comparison correction (GraphPad Prism). The difference between non-injected, invadolysin transcript only and both ATG-MO and Ex6 MO with invadolysin transcript (rescue) was not deemed to be statistically significant (*p*>0.05). Conversely, the difference between ATG-MO and all other experimental groups was considered most significant at positions A4 and T1-3 (*p*<0.01 to *p*<0.001). The difference between Ex6-MO and Ex6-MO rescue was deemed significant at T1and T2 (*p*<0.05), and T3 (*p*<0.001).

Confocal microscopy of the terminal neuromast clusters in a 48 hpf wild type fish revealed that invadolysin is localised in a circular pattern around the base of the neuromast (Supplementary Fig. 1A). In addition, invadolysin is observed to have a distinct nuclear periphery localisation in the surrounding cells. The hair cells and cell boundaries were observed by staining with an antibody to acetylated-tubulin.

48 hpf ATG-MO injected embryos were also analysed by confocal microscopy (Supplementary Fig. 1B). A significant reduction in the amount of invadolysin staining was observed. Despite the appearance of fewer neuromast clusters in the morphant fish, those that were present contained acetylated tubulin-positive hair cells. The Z-stacks for both samples were collected at 2.65 μm intervals; the images shown represent two sections approximately 16 μm apart.

### Invadolysin morphant fish exhibit melanophore distribution defects

**Fig. 4:**
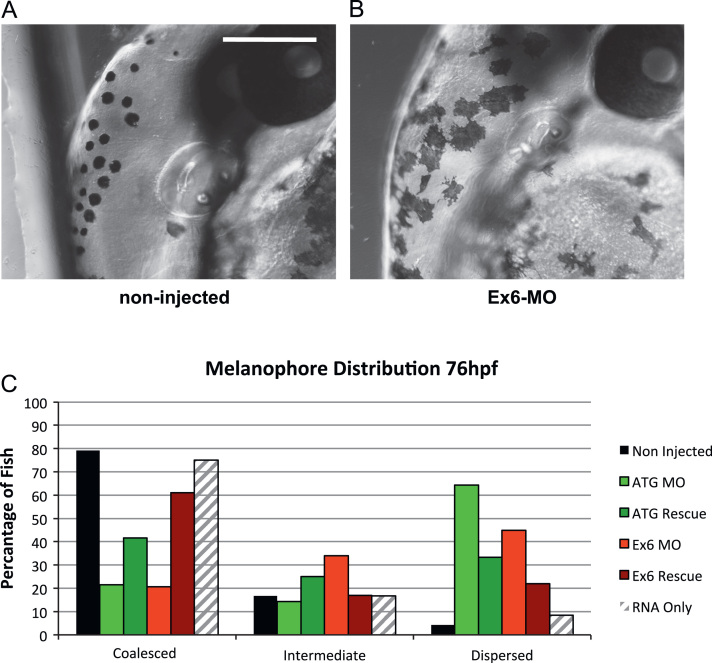
Comparison of melanophore appearance and distribution following invadolysin knock-down. (A) The coalesced appearance of melanophores on the head of a non-injected 74 hpf embryo. (B) Stellate appearance of melanophores on the head of an Ex6-MO injected embryo. Scale bar=250 μm. (C) Comparison of melanophore distribution over the yolk sack of samples from Fig. 3. A partial rescue of distribution phenotype is obtained by co-injecting full-length capped invadolysin transcript. A minimum of 24 fish were scored for each sample.

During the analysis of morphant phenotypes, we also observed defects in melanophore distribution after injection with morpholinos targeting invadolysin. Melanophores are mobile pigment cells which give the adult zebrafish its characteristic stripes. They arise from the neural crest, become pigmented, and then migrate to four distinct compartments of the developing embryo: directly above and below the yolk sac, along the median line of the trunk and along the dorsal plane [22]. While the melanophore cells are migrating, they exhibit a striking stellate appearance, which is maintained until the cells coalesce at the appropriate body location. They then retract their projections and exhibit a more rounded appearance (Fig. 4A). The melanophores in fish injected with either the ATG-MO or Ex6-MO continue to exhibit a stellate appearance for longer than in non-injected control fish (Fig. 4B). In addition, the movement to appropriate body compartments appears to be hindered. The migration of melanophores was scored by monitoring the position of the cells across the yolk sac (Fig. 4C). Cells which had moved toward the appropriate regions of the developing fish were scored as ‘coalesced’, while cells that still remained stellate and spread across the yolk sac were considered ‘dispersed’. Those cells mid-way in position were classed as ‘intermediate’. At 76 hpf, 79% of non-injected fish showed melanophores coalesced at the appropriate body location (black bars), whereas only 21% of both the ATG-MO and Ex6-Mp injected fish displayed this distribution (green and red bars). The majority (64% and 45%) of the ATG-MO and Ex6-MO injected fish exhibited a dispersed melanophore distribution, a pattern that was only observed in 4% of non-injected control fish. Both non-injected and ATG-MO injected fish showed a similar intermediate distribution pattern of 17% and 14% respectively, whereas 35% of the Ex6-MO injected fish exhibited an intermediate pattern.

Melanophore distribution following co-injection of both ATG-MO and Ex6-MO with full-length invadolysin transcript was analysed to determine whether this phenotype could also be rescued. Fish injected with invadolysin transcript alone displayed a similar melanophore distribution to non-injected fish with 75% exhibiting a coalesced pattern and 8% exhibiting the dispersed pattern (compare black and striped bars). However, 42% of fish co-injected with ATG-MO and invadolysin transcript (dark green bars) and 61% of fish co-injected with Ex6-MO and invadolysin transcript (dark red bars), displayed coalesced melanophores, suggesting this phenotype can also be partially rescued by co-injection of invadolysin transcript.

It should be noted that rescue experiments were performed with full-length invadolysin transcript, and the target sequence of the ATG-MO would be present in this construct, and might conceivably result in a depletion effect on the injected transcript as well as on the endogenous mRNA. However, the Ex6-MO only contains a 5 nucleotide over-lap of exon sequence, and should therefore not target the injected invadolysin transcript. Thus, we have confidence in our rescue after comparing the similar effects of ATG-MO and Ex6-MO data.

Two-way analysis of variance showed no statistical difference (*p*>0.05) with respect to melanophores classed as intermediate between all groups and no statistical difference between non-injected and invadolysin transcript-only injected fish. The difference between non-injected and both ATG-MO and Ex6-MO injected fish was considered to be highly significant (*p*<0.001) for both coalesced and dispersed melanophore localisations. Comparison of non-injected with rescue fish showed no significant difference (*p*>0.05). Therefore, we conclude that the co-injection of invadolysin transcript with either the ATG-MO or Ex6-MO is able to at least partially rescue the morphant phenotype caused by injection of either MO alone.

### INV is a highly dynamic protein

**Fig. 5:**
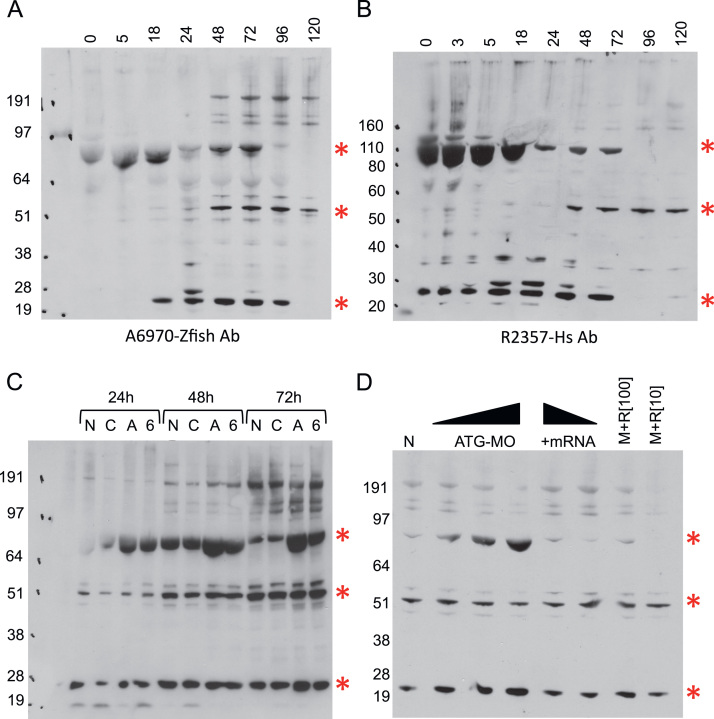
Analysis of Invadolysin by immunoblotting. (A) Immunoblot analysis on zebrafish extracts obtained at 0–120 hpf probed with an antibody raised against a peptide specific to zebrafish invadolysin. (B) Immunoblot analysis on zebrafish larval extracts obtained at 0–120 hpf probed with an antibody raised against the *C*-terminal half of human invadolysin. (C) Immuno-blot analysis on zebrafish larval extracts obtained from non-injected (*N*), Control (*C*), ATG-MO (A), and Ex6-MO (6) injected embryos at 24, 48 and 72 hpf, probed with zebrafish invadolysin antibody. (D) Immunoblot analysis on zebrafish extracts obtained from non-injected (*N*), ATG MO injected exhibiting low, medium and high amount of morpholino fluorescence (ATG-MO), decreasing amounts of injected RNA alone (R1 & R2), or co-injected with ATG-MO and invadolysin transcript (M+R1/R2). Molecular weight markers are shown in kDa, red asterisks denote bands of 74, 55 and 22 kDa.

As described above, we generated two rabbit poly-clonal antibodies against a peptide from zebrafish. We characterised these antibodies by both immunofluorescence and immunoblotting. While immunofluorescence shows temporal specific localisation of the protein (Fig. 2B–E), immunoblotting also displayed intriguing temporal changes in the forms of invadolysin detected over time (Fig. 5A). From 0–72 hpf, a band of 74 kDa, predicted to represent the full-length protein, is observed in all samples. A lower molecular weight band of 22 kDa is also detected in the 18, 24, 48, 72 and 96 hpf samples. In addition, a band of 55 kDa is observed in the samples from 48–120 hpf. When the same samples were probed with an antibody raised against the C-terminal half of human invadolysin, a similar pattern was observed (Fig. 5B). These results suggest that the three most abundant bands on immunoblots represent different forms of the invadolysin protein.

**Fig. 6:**
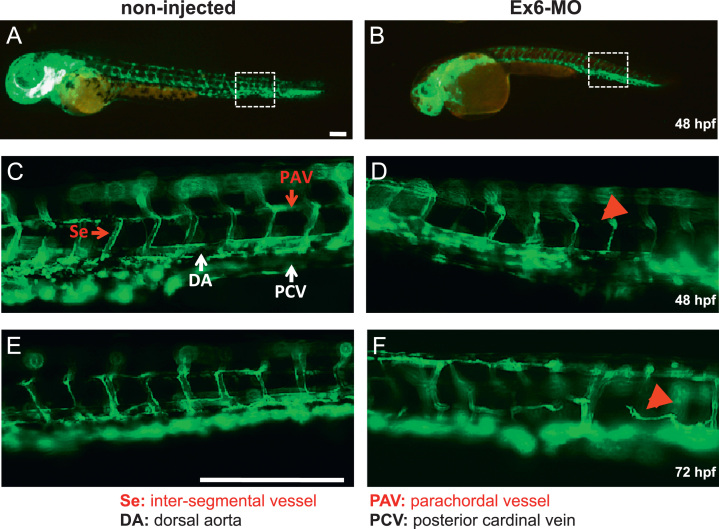
Formation of vessels arising via angiogenesis is perturbed in invadolysin morphant fish. Comparison of vascular formation in non-injected (A, C and E) and Ex6-MO injected (B, D and F) *Tg*(*fli*1:*egfp*) fish at 48 and 72 hpf. The dorsal aorta (DA) and posterior cardinal vein (PCV) formed through vasculogenesis appear normal in both non-injected and Ex6-MO injected fish. However, the inter-segmental vessels (Se) and parachordal vessels (PAV) generated by angiogenic sprouting are either poorly formed or absent in the Ex6-MO injected fish at 48 and 72 hpf (D and F, arrowheads). Scale bar=250 μm.

When morphant extracts (ATG-MO [A] and Ex6-MO [6] injected fish) were compared to control extracts (non-injected [N] and Con-MO [C] injected fish), we observed that the 74 kDa band accumulated at all time points (Fig. 6C). This was surprising, as one might expect a simple decrease in the amount of protein, and may instead suggest an auto-regulatory, or self-processing role for invadolysin. When samples from 76 hpf non-injected fish were compared to ATG-MO injected fish that exhibited a low, medium and high level of incorporated morpholino, the amount of 74 kDa protein (which we deem to be full-length) was observed to accumulate in the morphant fish (Fig. 6D, lanes 1, 2, 3 & 4). Additionally, when fish that had been injected with invadolysin transcript alone (Fig. 6D, R1 [100 ng] and R2 [10 ng]) were compared to those co-injected with ATG MO and mRNA, the samples showed very little difference to the non-injected control (Fig. 6D, M+R1 & M+R2).

### Invadolysin knock-down does not impede an inflammatory response

We have previously shown that invadolysin was localised to the leading edge of migrating human macrophages, and was required for gonad formation in the *Drosophila* embryo [1], a process highly dependent on active cell migration. Therefore, it was compelling that two different events during zebrafish development requiring cell migration—neuromast deposition and melanophore distribution—were also affected upon disruption of invadolysin. To further elucidate invadolysin's role in cell migration and to ask whether specific pathways dependent on particular chemokine/receptor combinations were affected, we examined the migration of cells involved in an inflammatory response following the knock-down of invadolysin.

The chemokines CXCL1-8, along with the CXCR1 and CXCR2 receptors, are involved in inflammatory migratory pathways [23]. We therefore injected *Tg*(*mpx:eGFP*) fish (GFP expressed under the myeloperoxidase promoter) with invadolysin morpholinos. This GFP reporter is expressed in circulating neutrophils (or heterophils) [24]. Non-injected and ATG-MO injected embryos were allowed to develop for 48 h and then subjected to a “tail trans-section” wounding assay [24]. By quantifying the number of neutrophils that migrated towards the wound at 5.5 h and then away at 24 h (resolution), an inflammatory response could be assessed (Supplementary Fig. 2). Although the number of circulating neutrophils in the ATG-MO injected fish was significantly lower than in the non-injected control fish (*p*<0.001), the GFP-expressing neutrophils in the morphant fish were still able to migrate to the tail-transected wound, and also to recover in a timely fashion to this wounding. We conclude therefore that invadolysin is unlikely to play an active role in the CXCL1–8 and CXCR1/CXCR2 pathway important for this inflammatory response. Whether invadolysin plays a role in determining the number of circulating neutrophils is currently under investigation.

### INV morphant fish exhibit vascular defects

We wished to determine whether invadolysin plays a role in the dynamic process of vascular development as metalloproteases of other families, e.g. meprins and MMPs, have been shown to be required for this process in zebrafish [25,26]. The *Tg*(*fli*1*:egfp*) line originally generated by Lawson and Weinstein [27] provides an exquisite visual reporter system in which to examine both vasculogenesis (the *de novo* synthesis of vessels) and angiogenesis (the sprouting of new vessels from existing vessels).

Non-injected and Ex6-MO injected *Tg*(*fli*1*:egfp*) fish were analysed at 48 and 72 hpf (Fig. 6). At the whole larval level, the stereotypical grid-like pattern of vasculature development as described by Isogai et al. [28] was evident in non-injected animals, but was clearly aberrant in the Ex6-MO injected fish (Fig. 6A and B). At higher magnification, the main vessels that are generated via vasculogenesis - the dorsal aorta (DA) and the posterior cardinal vein (PCV), appeared to form normally in both non-injected fish and Ex6-MO injected fish (Fig. 6C and D). However, the inter-segmental vessels (Se) and parachordal vessels (PAV), which are normally formed through angiogenic sprouting, are either poorly developed or absent in the Ex6-MO injected fish at both 48 and 72 hpf (Fig. 6D and F).

**Fig. 7:**
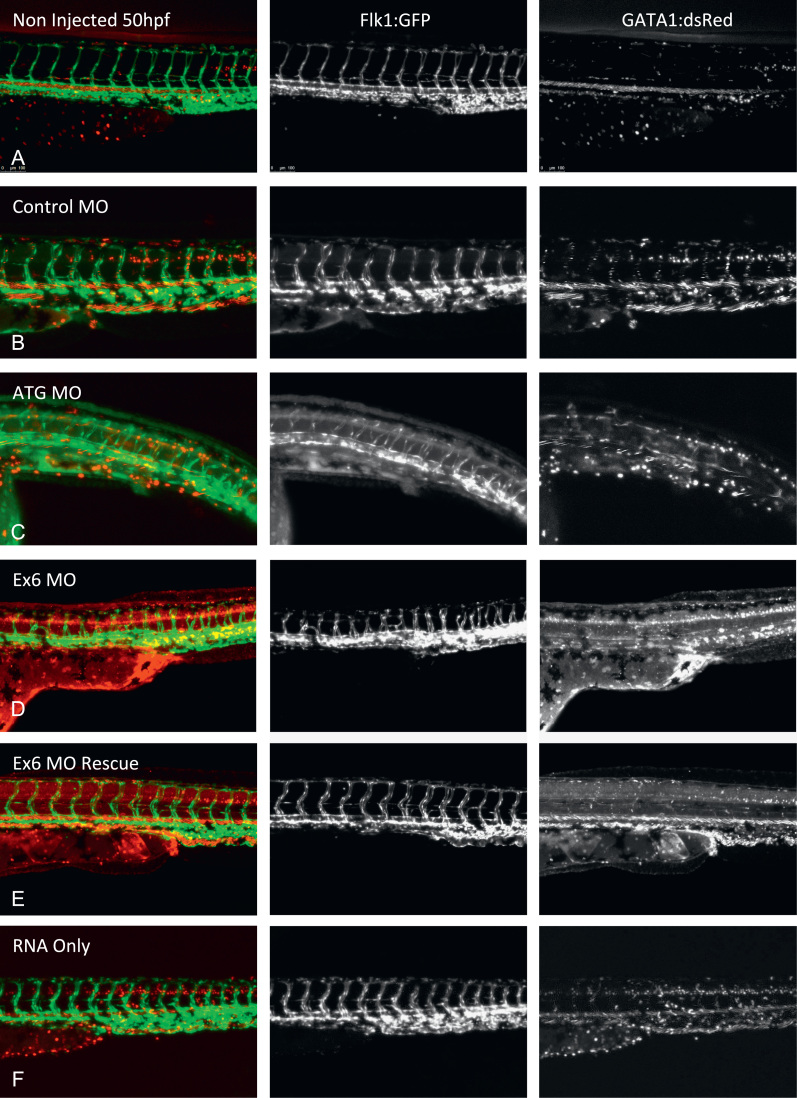
Comparison of vascular formation and blood flow in knock-down and control fish. Single images from time lapse confocal microscopy visualising vascular formation and blood flow in *Tg*(*flk*1:*GFPnls*;*GATA*1:*dsRed*) non-injected (A), Con-MO (B), ATG-MO (C) Ex6-MO (D) Ex6-MO and transcript (E) and invadolysin transcript only (F) injected fish at 50 hpf. Scale bar=100 μm. Approximately 50 embryos were analysed per condition. Of the 33 surviving Ex6-MO and invadolysin transcript, 28 fish displayed a rescue phenotype (85%).

To further characterise the effect of invadolysin depletion on vascular development and to assess whether blood flow was also perturbed, we injected the double transgenic *Tg*(*flk*1:*GFPnls*;*GATA*1:*dsRed*) line with Con-MO, ATG-MO, Ex6-MO, Ex6-MO plus full length invadolysin transcript, and invadolysin transcript alone (Fig. 7A–F). The fish were then immobilised in 3% methylcellulose containing 4.2% MS222, and imaged by video confocal microscopy. As observed by *flk-*1:*GFPnls* expression in endothelial cells, fish injected with either ATG-MO or Ex6-MO exhibit impaired vascular development (Fig. 7C and D) in comparison to control fish (Fig. 7A, B and F). This impairment in vascular development was reversed by co-injection of full-length invadolysin transcript in 85% of the 33 embryos positively co-injected with Ex6-MO and transcript (Fig. 7E). *GATA*1:*dsRed* is expressed in erythrocytes, and permits visualisation of circulatory flow. The *GATA*1:*dsRed* expressing cells appear to move freely around the primary vessels in the ATG-MO and Ex6-MO knock-down fish, however, as anticipated were unable to pass through the under-developed architecture of the intersegmental and parachordal vessels.

The involvement of invadolysin in angiogenesis suggests in its simplest form, a functional aspect common to the morphant phenotypes of disrupted neuromast deposition, melanophore distribution, and angiogenesis. We can speculate that this involves the ability of cells to migrate in response to *specific* signalling cues. Therefore we hypothesise that invadolysin may play a fundamental role in regulating particular chemokine signalling pathways, e.g. those required for neuromast deposition, melanophore distribution and angiogenesis.

## Discussion

In this study, we demonstrate that a morpholino-induced knock-down of invadolysin in zebrafish embryos disrupts three distinct processes dependent on cell migration in developing zebrafish. These include neuromast deposition, melanophore distribution and angiogenesis. It is thus the first metalloprotease shown to be involved in all three of these critical and dynamic processes, and suggests a role for invadolysin in a mechanism shared by these morphogenetic events.

The developmentally conserved pathway along which the cells of the neuromast primordium migrate is defined by the expression of SDF1a (stromal-derived factor 1 alpha, or CXCL12—Human Gene Nomenclature Database). SDF1a is a potent chemo-attractant ligand, to which the CXCR4b and CXCR7 receptors respond. The relationship amongst SDF1a, CXCR4b and CXCR7 expression in teleost lateral line development has been the subject of intense recent research [29–35]. Zebrafish have two orthologues of both SDF1 and CXCR4 [36–40]; however, it is the interaction between SDF1a and CXCR4b that is necessary for the accurate migration of primordial cells and formation of the posterior lateral line. Whether CXCR7 acts independently of CXCR4b [33], or whether there is a negative interplay between the two receptors remains subject to debate [32].

SDF1 (or CXCL12) belongs to the CXC motif family of chemokines. CXCR4 and CXCR7 are G-protein coupled receptors (composed of seven trans-membrane domains) that respond to this chemokine. Both the SDF1a and CXCR4b molecules are involved in a variety of diverse cell migratory pathways in different model systems including migration of dentate granule cells in mouse [41], neural and sensory axon path-finding [42,43], and formation of muscle in zebrafish [17].

Of particular relevance to this study, SDF1a has been shown to play a direct role in the formation of a fish's stripes [22]. Svetic et al. demonstrated that when a bead soaked in recombinant human SDF1 is fixed directly on the surface of a 24 hpf embryo, by 48 hpf the surrounding melanophores exhibit a stellate appearance, migrate from their normal location, and accumulate at the site of ectopic SDF1. This work on SDF1 provides a crucial mechanistic link between the processes involved in both lateral line development and melanophore migration.

Intriguingly, the RNA in situ hybridisation pattern observed with an invadolysin-specific probe is very similar to the staining pattern observed for *myoD* expression [44], and also shows similarities to the expression pattern of *sdf*1*a, cxcr*4*a,* and *cxcr*4*b* during somitic myogenesis [17]. The localisation of invadolysin as ascertained by immunofluorescence to the developing olfactory system, the limb bud, tail fin and lateral line also suggests a role for invadolysin during these highly dynamic developmental stages.

By examining the neutrophil inflammatory response in the tail transection assay, we were able to demonstrate that although the number of *mpx:eGFP* positive cells was decreased in the invadolysin morphant fish, the response and recovery mechanisms to injury were still intact. As the CXCL1–8 ligands and CXCR1/CXCR2 receptors are the signalling molecules involved in the neutrophil inflammatory response [23], we conclude that invadolysin does not play an active role in this pathway. We therefore propose that invadolysin functions in the SDF1a/CXCR4b pathway which has been shown to play a role in both neuromast and melanophore cell migration [22,29,31,32,45].

Previous work in our lab has shown that mutation of *invadolysin* in *Drosophila* inhibits primordial germ cell migration in developing embryos. Although obvious homologues of SDF1a, CXCR4 and CXCR7 are not apparent in *Drosophila*, the G protein-coupled receptor “Trapped in endoderm 1″ (TRE1) is required for primordial germ cell migration across the posterior mid-gut [46]. TRE1 is also involved in the appropriate polarisation and dispersal of primordial germ cells [47] (reviewed in [48]).

Is there also a link between SDF1a and angiogenesis? Angiogenesis is the formation of new vessels from pre-existing vessels and is required for the establishment of a fully functional circulatory system [49]. Sprouting of endothelial cells to form blood vessels involves the competitive selection of a leading tip cell, which is regulated by delta-like 4 (Dll4)-Notch signalling and vascular endothelial growth factor (VEGF) [50–55]. SDF1a and CXCR4 have been implicated in angiogenesis—in particular with the processes that facilitate the progression of cancer, such as tumour cell proliferation, metastasis and angiogenesis [56–66]. Not surprisingly, the suitability of SDF1a and CXCR4 as potential targets for anti-cancer therapeutics is the subject of on-going research [67–69].

In addition to chemokines and other signalling molecules, a number of proteases have also been identified as playing roles in angiogenesis: these include meprins [25] and matrix metalloproteases (MMPs), in particular MMP2, MMP-9 and MT1-MMP (also called MMP-14) [70,71], reviewed more recently [72]. The role of MMPs in angiogenesis is to enable endothelial tip cells to branch and form lumen tubes through degradation and remodelling of the local extracellular matrix [26]. These lumen tubes are then stabilised by the recruitment of support cells and pericytes [73] that in turn become new blood vessels. MMP activity is inhibited by TIMPs (Tissue Inhibitor of MetalloProteases), which have also been shown to regulate angiogenesis, wound repair and tumour metastasis (recently reviewed in [74]). What function invadolysin may have in the interplay with MMP or meprin proteases is currently unknown.

Although we have performed siRNA studies targeting invadolysin in HeLa and A375 cells, the level of protein was not dramatically affected (although invadolysin transcript level was decreased). One plausible explanation for this is that invadolysin is a particularly stable protein. In spite of this, invadolysin-depleted cells displayed a number of cell migration phenotypes including an impaired ability to degrade FITC-gelatin, and a reduced ability to ‘heal’ *in vitro* scratch wounds (data not shown).

## Conclusions

Taking into consideration the phenotypes observed upon invadolysin disruption in zebrafish, *Drosophila*, and siRNA treated human cells, there is compelling evidence for invadolysin's role in facilitating chemokine signalling and appropriate cell migration. Due to the observed effects on neuromast deposition, melanophore migration, and angiogenesis (but *not* on neutrophil migration after wounding), we believe that attention on the SDF1 signalling pathway is warranted. Detailed investigation is currently underway to further clarify the molecular mechanisms by which invadolysin exerts its influence.

## Author contributions

SV conceived and implemented experiments, and co-wrote the manuscript. MMSH conceived experiments and co-wrote the manuscript.

## Footnotes

Appendix A Supplementary data associated with this article can be found in the online version at 10.1016/j.yexcr.2013.02.005.
