# Supplementary figures and images for: Perturbation of invadolysin disrupts cell migration in zebrafish (Danio rerio)
Source: Exp Cell Res. 2013 May 1;319(8):1198–212. doi: 10.1016/j.yexcr.2013.02.005 (PMC3632754; doi:10.1016/j.yexcr.2013.02.005)

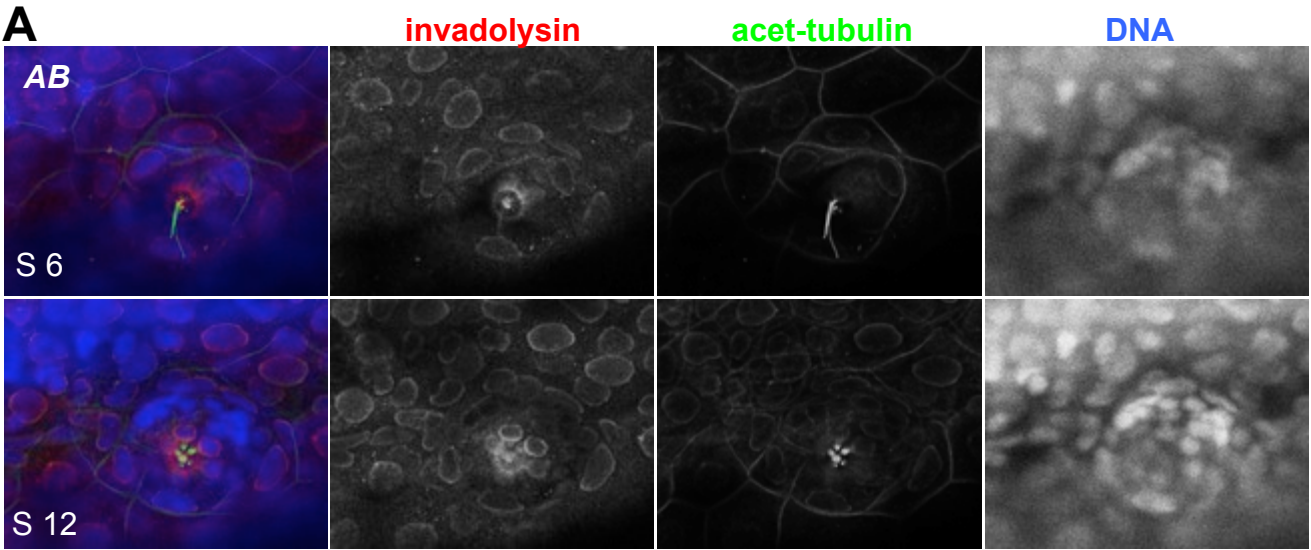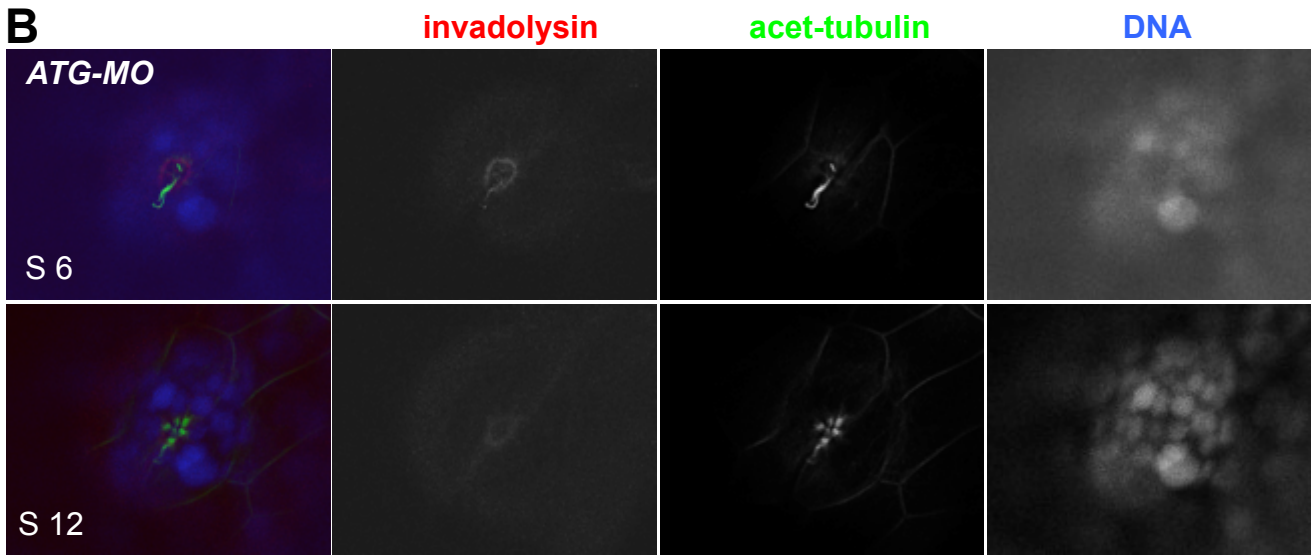

Supplement: Supplementary file 1 — Supplementary data [file mmc1.pdf]

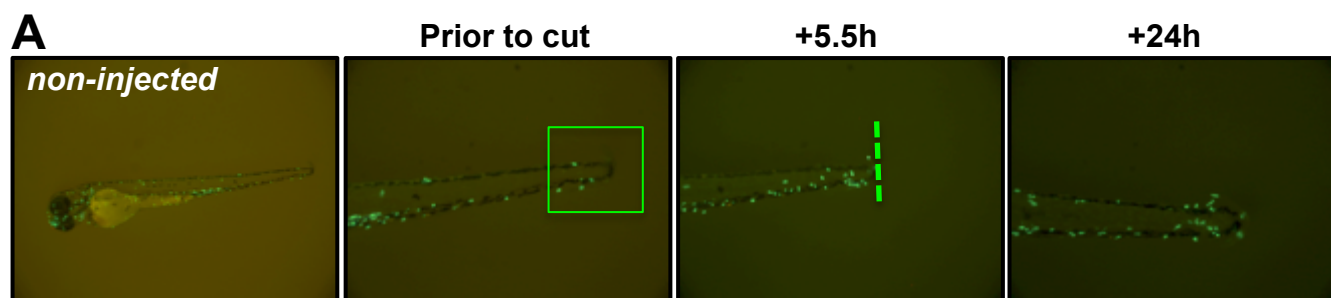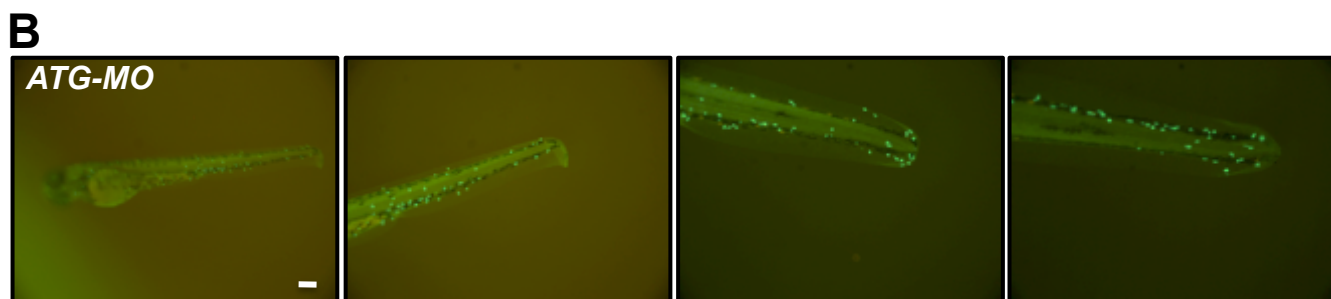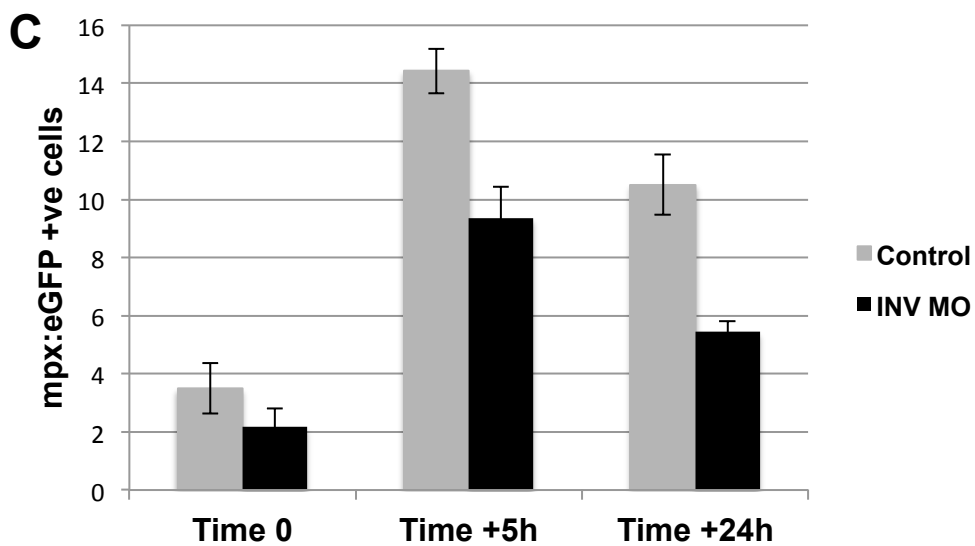

Supplement: Supplementary file 2 — Supplementary data [file mmc2.pdf]
